# Dihydromyricetin inhibits cell proliferation, migration, invasion and promotes apoptosis *via* regulating miR-21 in Human Cholangiocarcinoma Cells

**DOI:** 10.7150/jca.45970

**Published:** 2020-07-29

**Authors:** Lei Chen, Zhou-Sheng Yang, Yang-Zhao Zhou, Yang Deng, Pei Jiang, Sheng-Lan Tan

**Affiliations:** 1 Department of Pharmacy, The Second Xiangya Hospital, Central South University, Changsha, China, 410011.; 2 Institute of Clinical Pharmacy, Central South University, Changsha, China, 410011.; 3 Department of Pharmacy, The People's Hopital of Guangxi Zhuang Autonomous Region, Nanning, Guangxi, China, 530021.; 4 Department of Cardiovascular Surgery, The Second Xiangya Hospital, Central South University, Changsha, China, 410011.; 5 Department of Pharmacy, The Third Hospital of Changsha, Changsha, China, 410015.; 6 Department of Clinical Pharmacy and Pharmacology, Jining First People's Hospital, Jining Medical University, Jining, China, 272000.

**Keywords:** Dihydromyricetin, cholangiocarcinoma, miR-21, PTEN, Akt

## Abstract

Dihydromyricetin, the most abundant natural flavonoid isolated from Ampelopsis grossedentata, exhibits broad anti-tumor effects. However, the effects of dihydromyricetin on cholangiocarcinoma remain unclear. This study examined the anti-tumor effects of dihydromyricetin in two human cholangiocarcinoma cell lines HCCC9810 and TFK-1, and the underlying mechanism was also investigated. Our study was the first to show that dihydromyricetin significantly inhibited cell proliferation, migration, invasion and promoted apoptosis in cholangiocarcinoma cells. By analyzing the TCGA dataset, we found that expression of miR-21, an oncogene and a potential target of anticancer drugs for cholangiocarcinoma, was upregulated in cholangiocarcinoma tissues compared to paired control tissues. Moreover, dihydromyricetin significantly reduced the expression of miR-21 in a dose-dependent manner. Overexpression of miR-21 remarkably abolished the inhibitory effects of dihydromyricetin on cell proliferation, migration, invasion and abrogated its effect of promoting cell apoptosis in both HCCC9810 and TFK-1 cells. Dihydromyricetin remarkably increased the expression of PTEN and decreased the expression of phosphorylated Akt, while overexpression of miR-21 abrogated the modulation of PTEN/ Akt pathway by dihydromyricetin. Taken together, our study demonstrates that dihydromyricetin inhibits cell proliferation, migration, invasion and promotes apoptosis in cholangiocarcinoma cells via regulating miR-21.

## Introduction

Cholangiocarcinoma (CCA), derived from epithelial cells of the biliary tract, is the second most frequent primary hepatic malignancy 1. CCAs are divided into three categories as intrahepatic (iCCA), perihilar (pCCA), and distal (dCCA) according to their anatomical locations, and the international classification of CCA aggregates the pCCA and dCCA into one type. The optimal curative treatment for CCA is complete surgical resection; unfortunately, most patients are diagnosed as advanced-stage at presentation that is not amenable to surgery. The chemotherapy (gemcitabine plus cisplatin) is the first-line treatment for patients with advanced-stage CCA, irrespective of anatomical subtypes; however, the median overall survival is only less than one year 2. Thus, there is a tremendous need to seek new therapeutic agents for CCA therapy. In recent years, a number of natural products have aroused the interests of researchers for CCA treatment 3, 4. For instance, a recent study showed that genistein, an isoflavone rich in soybeans, could remarkably inhibit cell proliferation of two iCCA cell lines by inactivating epidermal growth factor receptor (EGFR) and protein kinase B (Akt) 3. Luteolin, a group of flavonoids distributed in vegetables and fruits, was also found to significantly decrease CCA cell viability and induce apoptosis 4.

Dihydromyricetin, the most abundant natural flavonoid and active compound in Ampelopsis grossedentata W.T. Wang (Vitaceae) used as herbal tea and traditional Chinese medicines for over hundreds of years in China, has been reported to show potent anti-tumor effects *in vitro* and *in vivo* studies 5, 6. For instance, dihydromyricetin combined with irinotecan chemotherapy remarkably delays the progression of colon cancer in mouse models 5. However, it has not been reported if dihydromyricetin exerts any anti-tumor effects in CCA yet.

MicroRNAs are short single‑stranded RNAs that play important roles in gene expression regulation at the post‑transcriptional level in many diseases including cancers 7. MicroRNA-21 (miR-21) is an oncogene in various types of human tumors. Recent studies indicate that miR-21 may be a potential diagnostic and prognostic biomarker for CCA 8, 9. As our previous study found that dihydromyricetin had great anti-atherosclerosis effects though regulating miR-21 10, we were interested to investigate whether dihydromyricetin could exert anti-tumor effects in human CCA cell lines, and whether the underlying mechanism was through regulating miR-21. Our study might provide a possible strategy for the treatment of CCA.

## Materials and Methods

### Cell culture and treatment

Human CCA cell lines HCCC9810 and TFK-1 respectively derived from intrahepatic and extrahepatic bile duct carcinomas were purchased from American Type Culture Collection (ATCC, USA) and maintained in RPMI 1640 medium (Thermo Fisher, USA) supplemented with 10% fetal bovine serum (Gibco, USA). Dihydromyricetin (Sigma-Aldrich, USA) was dissolved in dimethyl sulfoxide (Sigma-Aldrich, USA) to treat cell lines. Different concentrations of dihydromyricetin were tested and 150 μM was finally selected for treatment.

### Cell viability assay

The effects of dihydromyricetin treatment on cell viability were assessed using a Cell Counting Kit-8 (CCK-8) (Dojindo, Japan) assay. Briefly, cells were pre-cultured in 96-well plates and exposed to different concentrations of dihydromyricetin for 24 hrs. Then cell culture medium was replaced with 10 μL CCK-8 solution in each well, and cells were incubated for 1 h at 37 °C. The absorbance of the solution was measured at 450 nm using a Microplate Reader (Bio-Rad, USA).

### Cell proliferation assay

Cell proliferation was examined by the EdU assay (Beyotime, China). After treatments, cell culture medium was replaced with fresh medium containing 10 μM EdU and cells were incubated for 3 hrs. Then cells was fixed in 4% Paraformaldehyde for 15 min and incubated in 0.3% Triton-X 100 for 15 min, followed by Click buffer incubation for 30 min in dark at 37 °C and counterstained with Hoechst for 10 min. Finally, EdU-positive cells, DAPI-labeled cells and their merged images were captured under a fluorescence microscope (Zeiss, Germany).

### Cell apoptosis assay

Cell apoptosis was assessed by using the flow cytometry assay (BD, USA). After treatment, cells were collected by centrifugation, followed by washed twice with cold PBS and suspended in 200 μL binding buffer using the Annexin V-FITC/PI apoptosis detection kit (KeyGEN, China). Afterward, cells were stained with 2 μL Annexin V-FITC and 2 μL PI for 15 min in dark at room temperature. Finally, these cells were analyzed using a flow cytometer (BD, USA) and data were analyzed using the FlowJo software (Treestar, USA).

### Cell invasion and migration and assays

Cell invasion and migration were detected by using the Transwell assay with a pore size of 8 μM (Millipore, USA). For cell invasion, Transwell chambers with BD MatrigelTM Matrix (BD, USA) were used. After treatment, cells were re-suspended in the upper chambers with no-serum medium, and the lower chambers were supplemented with medium containing 10% FBS. After incubated for 24 hrs, cells on the lower side of the filter were fixed with 4% paraformaldehyde for 15 min and stained with 0.4% crystal violet (Merck, Germany) for 15 min. Finally, cells were counted and photographed under microscope. The method of cell migration was similar, except using chambers without BD Matrigel.

### RNA isolation and quantitative real-time polymerase chain reaction

Total cellular RNA was extracted using the RNAiso PLUS reagent kit (Takara, China). The commercial Reverse Transcribed kit (Takara, China) was used to synthesize cDNA, and real time qPCR was conducted using the TB Green real-time PCR kit (Takara, China). U6 was used as an internal control. The primers for miR-21-5p and U6 were ordered from RiboBio (Guangzhou, China).

### Cell transfection

HCCC9810 cells and TFK-1 cells were transfected with miR-21 agomir or agomir negative control (NC) (RiboBio, China) at a final concentration of 150 nM following the manufacturer's protocol. Cells were collected 24 hours post-transfection. Real time qPCR was used to confirm that miR-21 expression was specifically upregulated after transfection.

### Western blot assay

Cellular proteins were lysed with the radio immunoprecipitation assay (Beyotime, China). Proteins were separated by sodium dodecyl sulfate-polyacrylamide gel electrophoresis (SDS-PAGE) using a 10% gradient gel and transferred onto 0.45 μm polyvinylidene fluoride (PVDF) membranes. After blocked in 5% non-fat milk for 1 h, membranes were incubated with primary antibodies at 4 °C overnight and then secondary antibodies for 1 h at room temperature. Band exposure was achieved by electrochemiluminescence (ECL) (Beyotime, China) and band quantification was analyzed using the Image Lab™ Software (Bio-Rad, USA).

### Statistical analysis

All data were presented as mean ± standard deviation. Each experiment was performed in triplicates for at least three times. Statistical analyses were performed using the SPSS 22.0 statistical software (SPSS, USA). Comparisons among multiple groups were performed using a one‑way analysis of variance (ANOVA). P<0.05 was considered statistically significant.

## Results

### Dihydromyricetin inhibited cell proliferation and induced cell apoptosis in human CCA cells

**Figure 1:**
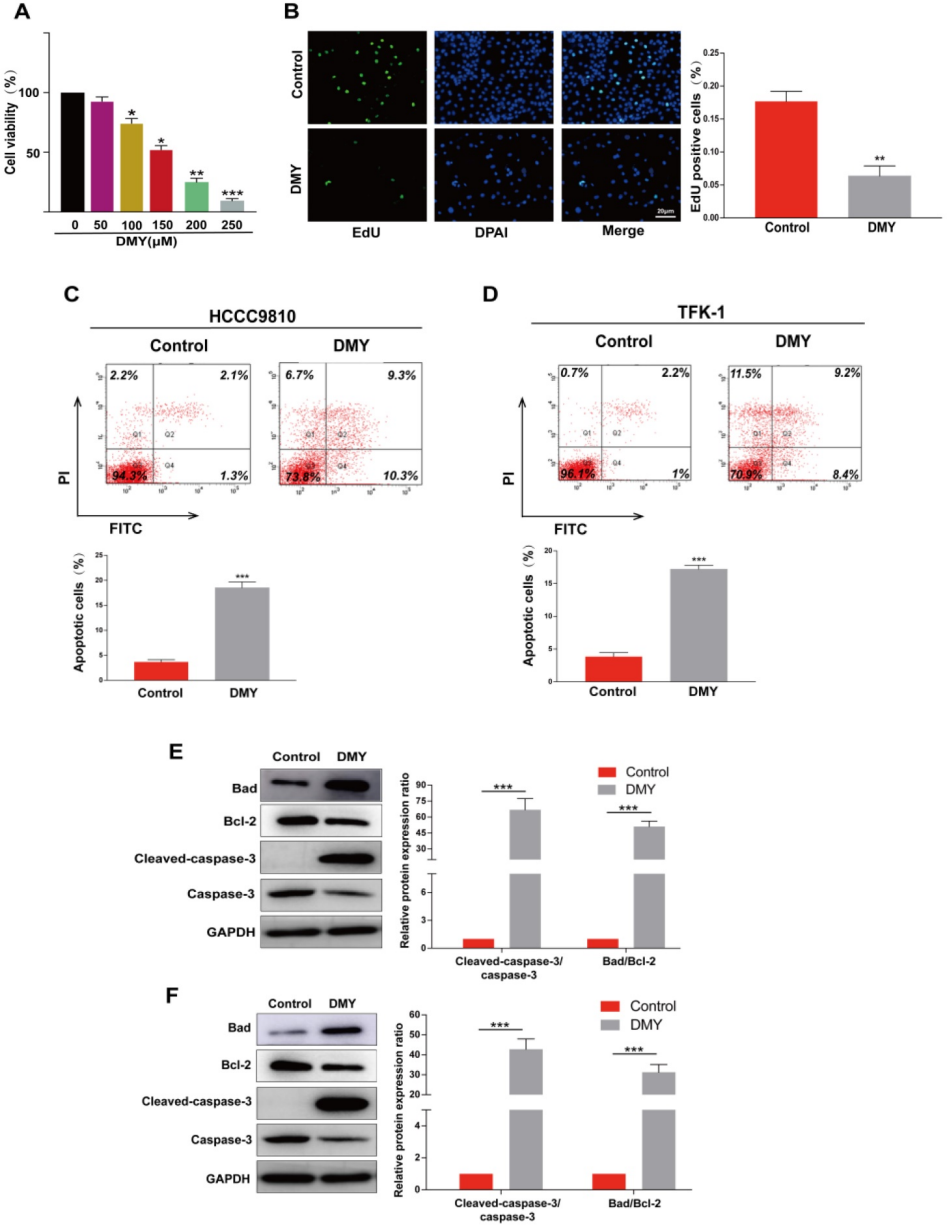
** Dihydromyricetin inhibits cell proliferation and promotes apoptosis in cholangiocarcinoma cells.** (**A**) CCK-8 assay for assessing cell viability in HCCC9810 cells. (**B**) EdU assay for assessing cell proliferation in HCCC9810 cells. (**C and D**) flow cytometry analysis for evaluating cell apoptosis in HCCC9810 and TFK-1 cells. (**E and F**) western blot for assessing the expressions of related proteins. All values are presented as the mean ± SEM, *P < 0.05, **P < 0.01, ***P < 0.001.

HCCC9810 cells were treated with dihydromyricetin at different concentrations for 24 hrs, and then cell viability was measured by the CCK-8 assay. Figure 1A showed that dihydromyricetin inhibited HCCC9810 cell viability in a dose-dependent manner, and the half maximal inhibitory concentration (IC50) value of dihydromyricetin was 156.8 μM, thus 150 µM of dihydromyricetin was selected in the subsequent experiments. Dihydromyricetin inhibited about 60% of cell proliferation (Figure 1B). The effects of dihydromyricetin on cell apoptosis and apoptosis-associated proteins were respectively evaluated by flow cytometry and western blot in both HCCC9810 and TFK-1 cells. As shown in Figure 1C and Figure 1D, 150 μM of dihydromyricetin treatment increased about 4 times of cell apoptosis compared with controls in both of the two human CCA cells. The results in Figure 1E and Figure 1F indicated that dihydromyricetin significantly promoted expression of cleaved-caspase-3 and the proapoptotic protein Bad as well as inhibited expression of the anti-apoptotic protein Bcl-2 compared to the control cells. These results indicate that dihydromyricetin could remarkably inhibit cell proliferation and promote cell apoptosis through the mitochondrial apoptotic signaling pathway in human CCA cells.

### Dihydromyricetin inhibited cell migration and invasion in CCA cells

**Figure 2:**
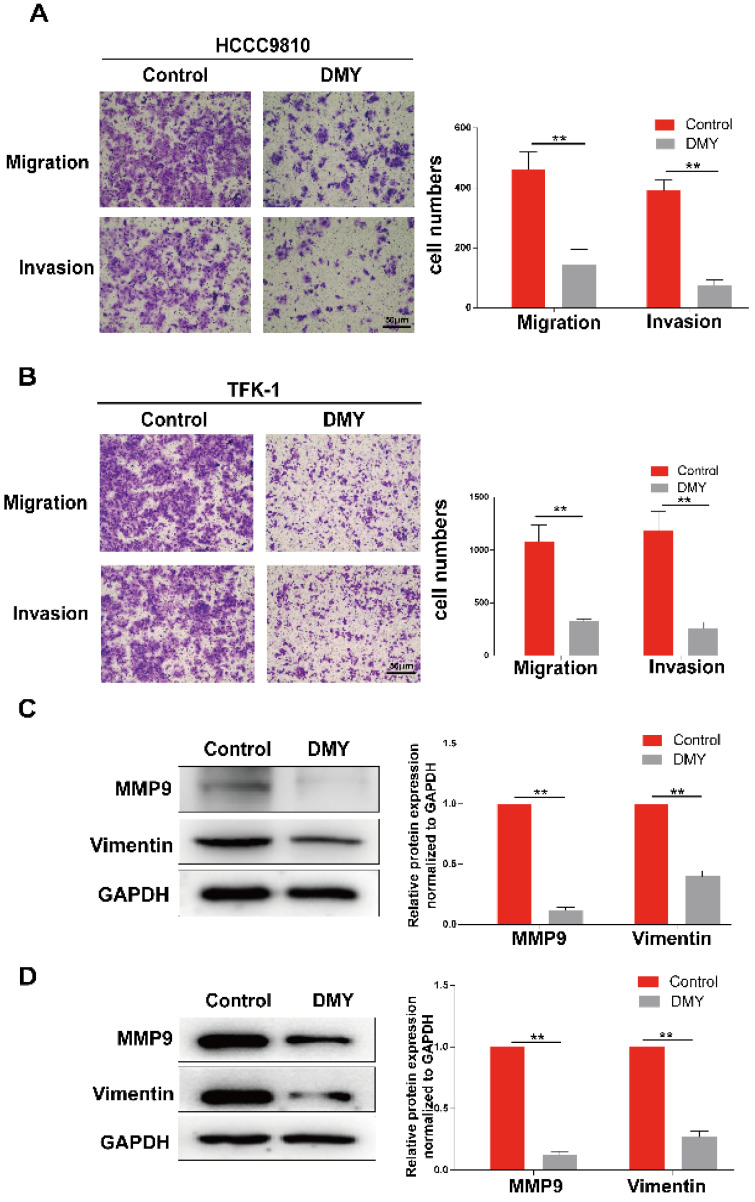
** Dihydromyricetin inhibits cell migration and invasion in cholangiocarcinoma cells.** (**A and B**) Transwell assay for evaluating cell migration and invasion in HCCC9810 and TFK-1 cells. (**C and D**) western blot for assessing the expressions of related proteins. All values are presented as the mean ± SEM, *P < 0.05, **P < 0.01.

Since CCAs are aggressive tumors often accompanied with metastasis, we evaluated the effects of 150 μM dihydromyricetin on cell migration and invasion by the Transwell assay in both HCCC9810 cells and TFK-1 cells in the next experiment. As shown in Figure 2A, cell migration and invasion in the HCCC9810 cells were suppressed respectively 60% and 80% in dihydromyricetin treated cells compared to vehicle treated control cells. Similarly, cell migration and invasion were also remarkably inhibited by dihydromyricetin in the TFK-1 cell lines (Figure 2B). The migration and invasion related protein levels of MMP9 and vimentin were significantly decreased by dihydromyricetin treatment in both HCCC9810 cells and TFK-1 cells as measured by Western Blot (Figure 2C and Figure 2D). These results indicate that dihydromyricetin could significantly inhibit cell migration and invasion.

### Expression of miR-21 is increased in human CCA, and dihydromyricetin reduced the expression of miR-21 in CCA cells

**Figure 3:**
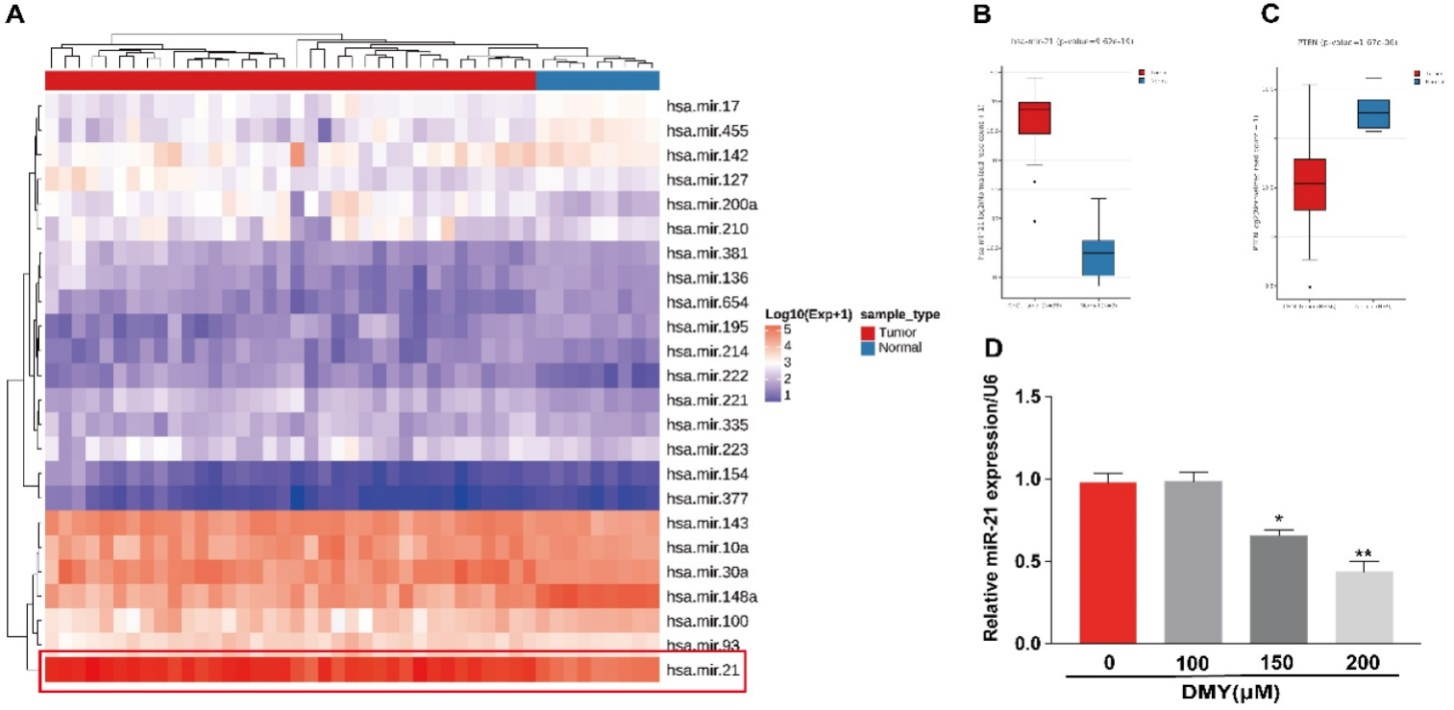
** Expression of miR-21 and PTEN in human cholangiocarcinoma tissue and dihydromyricetin reduces the expression of miR-21 in HCCC9810 cells.** (**A-C**) TCGA dataset for analyzing the expression of miR-21 in human cholangiocarcinoma. (**D**) Real time qPCR for assessing the impacts of dihydromyricetin on the expression of miR-21. All values are presented as the mean ± SEM, *P < 0.05, **P < 0.01.

Multiple studies have suggested that miR-21 plays an important role in the pathogenesis of CCA 9. Increased expression of miR-21 was associated with poor overall survivals 8, 9. Therefore, we analyzed the TCGA dataset using an online program (http://aipufu.com/index.html/) to investigate the expression of miR-21 in human CCA (Figure 3A). As shown in Figure 3B, the expression of miR-21 in human CCA tissues was significantly up-regulated compared to the non-tumor tissues. Moreover, phosphatase and tensin homolog (PTEN), the direct target of miR-21 and a tumor suppressor gene, was remarkably upregulated in human CCA tissues compared to the non-tumor tissues (Figure 3C). The results in Figure 3D demonstrated that the expression of miR-21 was inhibited by dihydromyricetin in a dose-dependent manner. These data suggest that the mechanism of dihydromyricetin's anti-cancer effects in CCA may be through negatively regulating the expression of miR-21.

### Overexpression of miR-21 abolished the anti-cancer effects of dihydromyricetin in CCA cells

**Figure 4:**
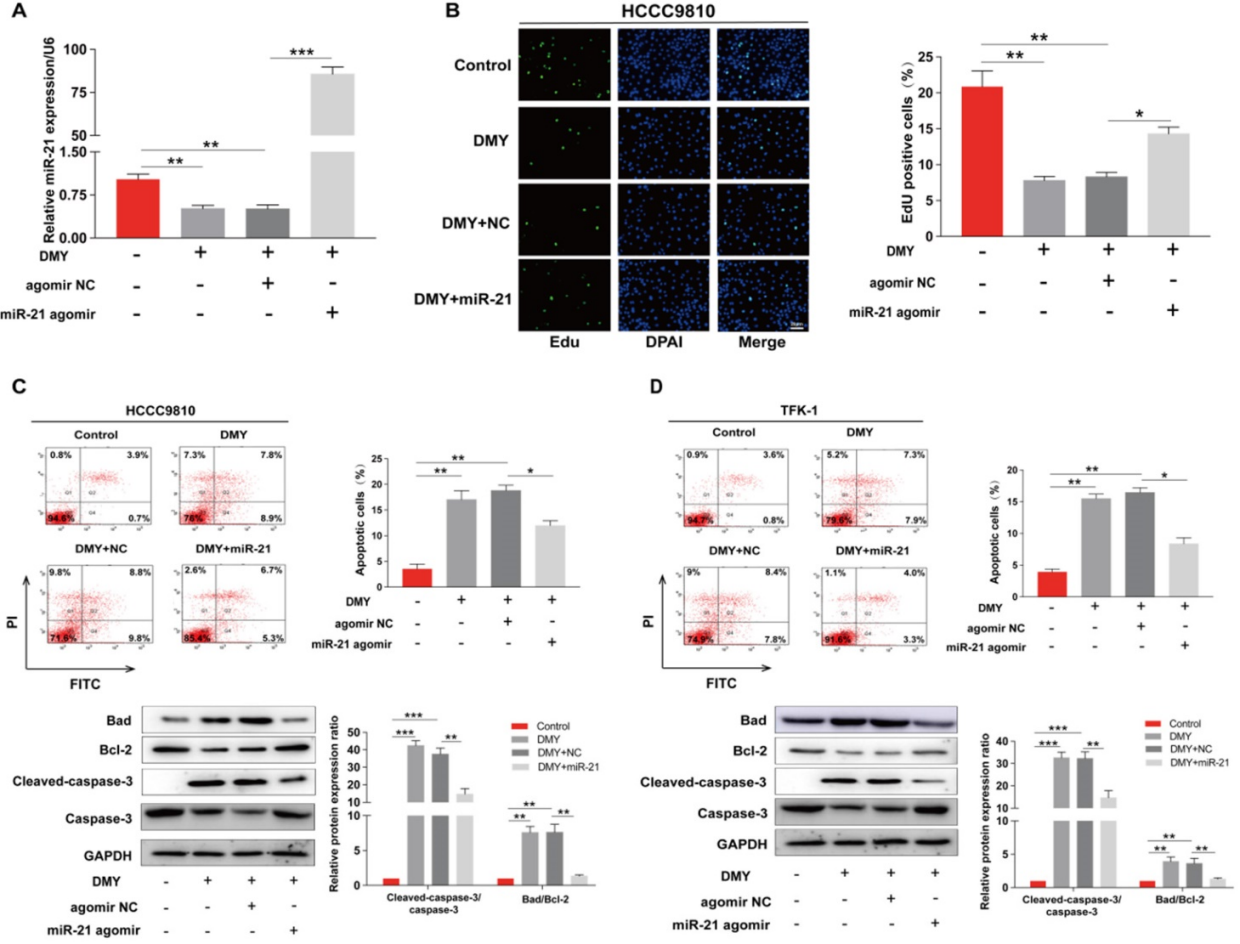
** Overexpression of miR-21 abolishes the inhibitory effect of dihydromyricetin on cell proliferation and promoting cell apoptosis.** HCCC9810 or TFK-1 cells were transfected with 100 nM miR-21 agomir or agomir non-specific control (NC), and then treated with or without 150 μM dihydromyricetin for 24 hours. (**A**) Real time qPCR analysis for assessing the relative expression of miR-21 in HCCC9810 cells. (**B**) EdU assay for assessing cell proliferation in HCCC9810 cells. All values are presented as the mean ± SEM, *P < 0.05, **P < 0.01, ***P < 0.001.

**Figure 5:**
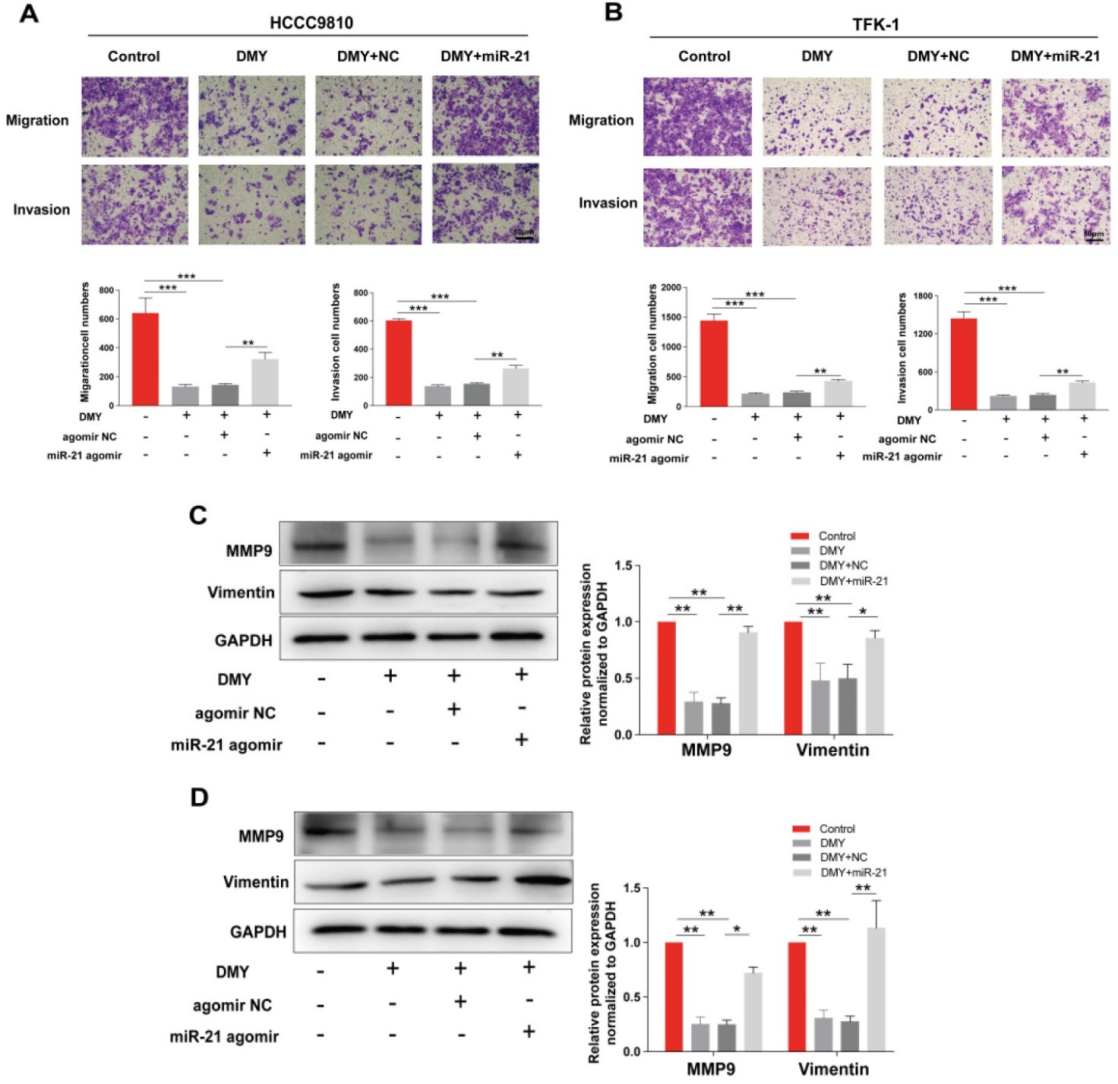
** Overexpression of miR-21 alleviates the inhibitory effect of dihydromyricetin on cell migration and invasion.** HCCC9810 or TFK-1 cells were transfected with 100 nM miR-21 agomir or agomir non-specific control (NC), and then treated with or without 150 μM dihydromyricetin for 24 hours. (**A and B**) Transwell assay for evaluating cell migration and invasion. (**C and D**) Western blot for assessing the expressions of related proteins. All values are presented as the mean ± SEM, *P < 0.05, **P < 0.01, ***P < 0.001.

In order to confirm the mechanism of the anti-tumor effects of dihydromyricetin in CCA cells is through negatively regulating miR-21, we transfected agomir NC or miR-21 agomir into HCCC9810 and TFK-1 cells. Figure 4A showed that the expression of miR-21 was remarkably up-regulated by the miR-21 agomir transfection compared to agomir NC transfection or control cells, indicating favorable transfection efficacy. Figure 4B showed that overexpression of miR-21 was able to partly abolish the inhibitory effect of dihydromyricetin on cell proliferation in HCCC9810 cells. Figure 4C and Figure 4D demonstrated that overexpression of miR-21 impaired the inducing effect of dihydromyricetin on cell apoptosis in both HCCC9810 and TFK-1 cells. As expected, protein levels of cleaved caspase-3 and the ratio of Bad to Bcl-2 were partly abrogated by miR-21 overexpression. Results in Figure 5A and 5B showed that overexpression of miR-21 partly abolished the inhibitory effect of dihydromyricetin on cell migration and invasion in both HCCC9810 and TFK-1 cells. Consistently, protein levels of MMP9 and Vimentin were significantly increased after overexpression of miR-21 compared to agomir NC transfected cells following treatment of dihydromyricetin. Taken together, these data showed that overexpression of miR-21 partly abolished the anti-tumor effects of dihydromyricetin in CCA cells, indicating the mechanism of the anti-tumor effects by dihydromyricetin is through regulating miR-21.

### Dihydromyricetin regulated the miR-21/ PTEN/ Akt pathway in CCA cells

**Figure 6:**
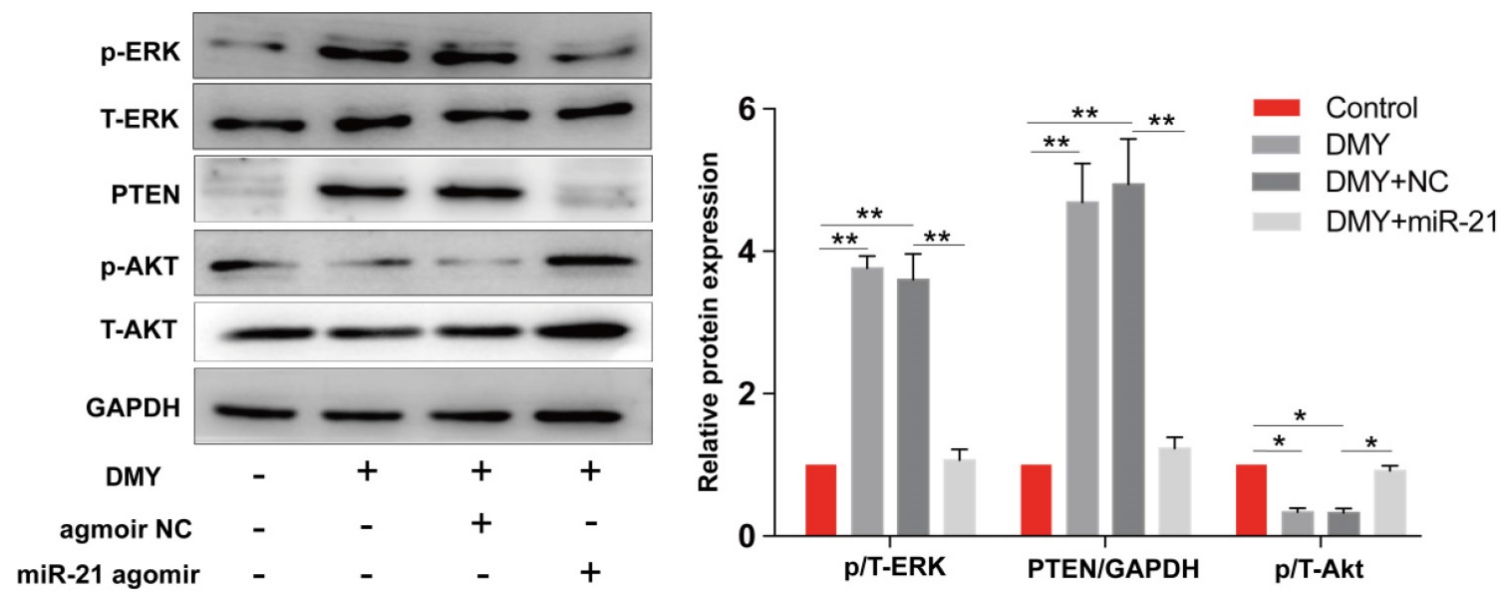
** Dihydromyricetin regulates the miR-21/ PTEN/ Akt axis in HCCC9810 cells.** HCCC9810 cells were transfected with 100 nM miR-21 agomir or agomir non-specific control (NC), and then treated with or without 150 μM dihydromyricetin for 24 hours. Western blot was used to determine the expressions of related proteins. All values are presented as the mean ± SEM, *P < 0.05, **P < 0.01.

The miR-21/PTEN/Akt axis is crucial to modulate cell apoptosis in multiple cancer cells 11, 12. Thus we further investigated the impacts of dihydromyricetin on the miR-21/PTEN/Akt pathway. As presented in Figure 6, compared to control cells, dihydromyricetin treatment remarkably upregulated the expression of PTEN and inhibited the phosphorylation of Akt (p-Akt) as determined by western blot. Lined with what we expected, overexpression of miR-21 decreased the expression of PTEN and increased the level of p-Akt. As mitogen-activated protein kinases 1/2 (ERK1/2) also play important roles in regulating cell apoptosis by suppressing the expression of Bad in the mitochondrial apoptotic signaling pathway, and the previous study showed that inhibition of miR-21 reduced phosphorylation of ERK in human colorectal cancer HCT116 cells 13, as a result, we further investigated the impacts of dihydromyricetin on the expression of ERK1/2. Contrary to our expectation, dihydromyricetin promoted the phosphorylation of ERK1/2 while overexpression of miR-21 decreased the expression of p-ERK1/2, which indicated that the mechanism of dihydromyricetin promoting apoptosis is not through regulating the ERK/ Bad pathway. In brief, our study confirmed that the anti-tumor effect of dihydromyricetin in CCA cells is through regulation of the miR-21/ PTEN/ Akt pathway.

## Discussion

Natural products have been attracting huge attention due to their broad biological activities with satisfying safety in human health care used as functional foods, health products or medicine. Dihydromyricetin, the main natural product isolated from the plant Ampelopsis grossedentata, has been widely investigated and shows a variety of biological functions, including ameliorating nonalcoholic fatty liver disease 14, anti-diabetes 15 and anti-atherosclerosis 10. In recent years, accumulating *in vitro* and *in vivo* studies have been conducted to explore its broad anti-tumor effects 5, 6. One study has shown that dihydromyricetin could inhibit cell growth and induce cell apoptosis in the hepatocarcinoma cells, and the underlying mechanism was through inhibiting the Akt/Bad signaling pathway and activating the mitochondrial apoptotic pathway 16. Considering the close anatomical position of the liver and biliary system, we were interested to explore whether dihydromyricetin also had anti-tumor effects in CCA. As expected, dihydromyricetin exerted significant anti-tumor effects in two human CCA cell lines manifesting as inhibiting cell proliferation, migration, invasion and promoting cell apoptosis (Figure 1 and 2). As dihydromyricetin had little cytotoxicity in human beings and modern pharmaceutics technology has helped to improve its solubility, stability and bioavailability, this natural product may be explored as a novel adjuvant agent for CCA treatment with further *in vivo* evidence.

It is well established that proliferation is crucial in tumor development and progression. In cancer cells, the signaling pathways controlling the proliferative response in normal cells are disturbed, resulting in the rapid growth of cancer cells that invade surrounding tissues and metastasize to distant sites 17. In this study, we found that 150 µM of dihydromyricetin suppressed about 60% of cell proliferation compared to control cells as evaluated by the EdU assay. Cancer cells are often resistant to apoptosis, a programmed cell death by which cells undergo death to restrict cell proliferation or in response to DNA damage. There are two major signaling cascades of apoptosis, including the extrinsic pathway such as tumor necrosis factor-related apoptosis and the intrinsic Bcl-2 family pathway launching the activation of caspases 9, 3 and 7 18. By using the flow cytometry assay, we showed that dihydromyricetin remarkably induced cell apoptosis in CCA cells. Western Blot showed that expressions of cleaved‑caspase‑3 and the Bad/Bcl-2 ratio were significantly up-regulated following dihydromyricetin treatment. These results indicate that regulating the intrinsic Bcl-2 family pathway is an important mechanism of dihydromyricetin inducing cell apoptosis, similar to previous studies in other cancer cells such as ovarian cancer and hepatocellular carcinoma 19, 20. Of note, a recent study showed that co-administration of Adriamycin (ADR) with dihydromyricetin significantly increased ADR-induced apoptosis, and the mechanism was not only related to the mitochondrial apoptosis but also associated with endoplasmic reticulum as the ROS levels and caspase-12 protein expression were increased 21. We will investigate if dihydromyricetin affects the endoplasmic reticulum related apoptosis in CCA in our further studies.

Rapid progression and metastasis is the huge obstacle in the treatment of CCA, thus reducing the capability of cancer cell invasion and migration may effectively restrain cancer metastasis. We then further investigated the effects of dihydromyricetin on cell invasion and migration in two CCA cell lines. By using the Transwell assay, we found that dihydromyricetin could significantly inhibit cell invasion and migration. As MMP9 expression was observed in 40% of extrahepatic, 56% of hilar and 62% of intrahepatic CCA, and MMP2 expression in CCA was uncommon (only 8% of CCA cases) 22, we examined the expression of MMP9 in our study. As shown in Figure 2, dihydromyricetin treatment inhibited the expressions of MMP9 and vimentin, the major mediators to regulate the processes of invasion and metastasis 23, 24. The results were similar to Zhang et al.'s report that dihydromyricetin inhibits cell migration and invasion through regulation of MMP9 expression in hepatoma cells 24.

Recently, a number of miRNAs including miR-424-5p 25 and miR-876 26 have been reported to regulate cell proliferation, apoptosis, migration and invasion in CCA. MiR-21 is considered as a potential diagnostic and prognostic biomarker for CCA 8, 9. The plasma level of miR-21 is significantly upregulated in patients with CCA compared to healthy population 8 and CCA patients with elevated expression of miR-21 were related to poor survivals 9. By analyzing the Cancer Genome Atlas (TCGA) database, we confirmed that the expression level of miR-21 in CCA tumor samples is significantly higher than that in paired normal tissue (Figure 3). So far, there has been no study to report that dihydromyricetin exerts the anti-tumor effect by modulating miRNA. However, recent studies by Yang et al. found that dihydromyricietin administration significantly restored the abnormal upregulation of miR-34a and ameliorated renal fibrosis in model mice 27. Moreover, our previous study has shown that dihydromyricetin attenuates TNF-alpha-induced endothelial dysfunction through regulating miR-21 in HUVEC cells 10. These studies indicate that regulating miRNA is an important mechanism of dihydromyricetin to exert its biological activities. Based on the above studies, we speculated that the mechanism of its anti-cancer effects in CCA was through regulating miR-21. In order to prove the hypothesis, firstly, we detected the levels of miR-21 after treatment of different concentrations of dihydromyricetin. As expected, the expression of miR-21 was inhibited by dihydromyricetin in a dose-dependent manner (Figure 3). Next, we conducted the rescue experiment in which overexpressed miR-21 by transfection with agomir in two CCA cell lines. In line with our speculation, overexpression of miR-21 partly abolished the anti-tumor effects of dihydromyricetin (Figure 4 and 5). These data indicated that the mechanism of dihydromyricetin exhibiting its anti-tumor effects is through modulating miR-21.

PTEN, a well‑known tumor suppressor involved in regulating cell growth and apoptosis in multiple cancers, is an important downstream target of miR‑21. miR‑21 inhibits the expression of PTEN by directly targeting its 3'-UTR 28. By analyzing the TCGA database, we showed that the expression of PTEN in CCA tumor samples is significantly lower than that in paired normal tissue, confirming that miR-21 negatively modulates the expression PTEN (Figure 3). Akt, which transduces signals from growth factors and oncogenes to downstream targets resulting in tumor development, is one of the most frequently hyperactivated signaling pathways in human cancers. The PTEN/Akt pathway has been reported to modulate cell apoptosis in multiple cancer cells. For instance, Zhu et al reported in CCA cells that upregulation of PTEN promotes cell apoptosis via downregulation of the Akt signaling pathway, while blockage of PTEN abolished the effects 29. Recently, several studies have reported that the miR-21/PTEN/Akt axis plays a significant role in modulating cell apoptosis 11, 12 in multiple cancer cells. Thus, inhibiting the miR-21/ PTEN/ Akt axis is an essential mechanism of endogenous or exogenous anti-cancer substances. In this study, we showed that dihydromyricetin treatment inhibited the expression of miR-21 and phosphorylated Akt and upregulated the expression of PTEN compared to control cells, while overexpression of miR-21 abolished the effect of dihydromyricetin on the expression of PTEN/ Akt (Figure 6). These data indicate that dihydromyricetin exerting the anti-tumor effects in CCA cells is through regulating the miR-21/PTEN/Akt axis and the miR-21/PTEN/Akt axis may be an important target for CCA treatment.

## Conclusion

Our study showed that dihydromyricetin suppressed cell proliferation, migration, invasion and promoted apoptosis through regulating miR-21 in cholangiocarcinomas cells. These findings suggest that dihydromyricetin exerts great anti-tumor effects in cholangiocarcinomas cells, yet further *in vivo* studies are required to verify our findings. Our study may provide a new sight into the treatment of cholangiocarcinomas.

## Abbreviations

CCA: cholangiocarcinoma
EGFR: epidermal growth factor receptor
CCK-8: Cell Counting Kit-8
NC: negative control
SDS-PAGE: sodium dodecyl sulfate - polyacrylamide gel electrophoresis
PVDF: polyvinylidene fluoride
ECL: electrochemiluminescence
IC50: half maximal inhibitory concentration
PTEN: phosphatase and tensin homolog
ERK1/2: mitogen-activated protein kinases
ADR: Adriamycin
